# Lipid droplet organized metabolic vulnerabilities in melanoma: from the MITF low state to ferroptosis sensitization

**DOI:** 10.3389/fmolb.2026.1948743

**Published:** 2026-09-30

**Authors:** Mengzhen Chen, Yi Wu, Muxin Zhao

**Affiliations:** The Second Hospital of Dalian Medical University, Dalian, Liaoning, China

**Keywords:** fatty-acid metabolism, ferroptosis, lipid droplets, lipid peroxidation, melanoma, metabolic vulnerability, MITF-low state, phenotypic plasticity

## Abstract

Melanoma’s ability to progress and develop resistance to therapies are the result of both genotypic changes and substantial levels of adaptability at the phenotypic and metabolic level. One of the most clinically relevant adaptive states is the microphthalmia associated transcription factor MITF low, which has been correlated with de-differentiation, invasion potential and drug tolerance, as well as altered redox status. As important regulators of this adaptation, lipid droplets have become involved in coordinating fat stores and mobilization, organelle signaling, membrane lipid composition and protection against oxidative stress. However, their role is context-dependent. For example, under certain melanoma conditions lipid droplets are able to isolate poly-unsaturated fatty acids (PUFA) from membrane phospholipids thus preventing ferroptosis. Under other conditions lipid droplet degradation through processes such as lipolysis, lipophagy or inter organelle lipid transfer results in the release of potentially oxidizable substrates increasing ferroptotic sensitivity. This review describes the impact of MITF-loss on melanoma cell lipid acquisition, desaturation, triglyceride synthesis and fatty acid oxidation, as well as phospholipid remodeling, antioxidant defense mechanisms and iron compartmentalization. An emphasis is placed on understanding the roles of several enzymes including DGAT1/2, SCD1, ACSL3, ACSL4, LPCAT3, MBOAT1/2, CPT1A, ACOX1, GPX4, FSP1 and lysosome-mitochondrial iron trafficking in defining whether melanoma cells are susceptible to or resistant to ferroptotic cell death. Additionally, this review provides an overview of clinical approaches that target protective lipid droplet programs, enhance membrane PUFA content, block compensatory fatty acid oxidation pathways and/or compromise ferroptosis-defensive systems. A major conclusion from this review is that the MITF low state is not uniformly ferroptotic; rather susceptibility depends upon a balance among lipid sequestration, membrane remodeling, oxidative metabolism, antioxidant defenses and cellular iron content. Identifying these state-specific metabolic requirements should provide a rationale for developing biomarker guided combinations of MAPK targeted therapy and immunotherapy-based treatments to selectively kill persistent populations of melanoma.

## 1 Introduction

In order to describe how melanoma adapts to drug treatment through reversible phenotypic changes, it is necessary to understand the role of reversible changes in cell identity, metabolism, and the ability of melanoma cells to tolerate stress. Immunotherapy using checkpoint inhibitors and targeted therapy against the BRAF-MEK pathway have significantly changed the treatment paradigm for melanoma, but durable long-term control continues to be limited by initial drug resistance, partial responses, and the existence of populations of drug tolerant cells that will develop stable drug resistance over time (Falcone et al., 2020). However, these remaining populations of cells do not exist based on genetic alterations alone. Melanoma cells exhibit a high degree of plasticity in their gene expression and metabolism based on a variety of stimuli including the presence of nutrients, levels of oxygen, levels of inflammation, the type of extracellular matrix they reside in, and other types of microenvironmental cues (Romano et al., 2021). This plasticity ensures the continued survival of viable subpopulations of melanoma cells and also necessitates the development of drugs targeting the adaptive state dependency of melanoma cells versus targeting the initiating mutation that drives the oncogenesis (Falletta et al., 2022).

Melanocyte specific transcription factors are key regulatory elements controlling melanocytic lineage identity and provide a conceptual framework for describing this plasticity. High levels of MITF (microphthalmia-associated transcription factor) usually support melanocytic differentiation, pigmentation, proliferation, lysosomal function, and selected antioxidant functions (Wessely et al., 2021). Conversely low levels of MITF are often associated with de-differentiation, invasiveness, inflammatory signaling and treatment resistance. Therefore, we no longer think about the earlier proliferative model as two separate states but instead see a continuum that now includes melanocytic, transitional, starved-like, neural crest-like and undifferentiated states (Huang F. et al., 2021). Thus MITF-low melanomas should not be treated as a single biological entity. Different MITF-low populations can lose melanocytic lineage identity while still having differences in RTK activity, lipid utilization, iron distribution and antioxidant defenses. Additionally recent studies show that lineage switchers like TFE3 and mechanically-induced chromatin remodeling regulate invasive behavior indicating that phenotype switches are controlled by multiple lineage and environmental signals (Pellerin et al., 2020).

Lipid metabolism plays a significant role in this process since fatty acids act as both structural components of membranes, energy substrates, signaling molecules, and sources of oxidative damage (Dikalov et al., 2024). As previously described MITF regulates the balance among saturated, mono-unsaturated and poly-unsaturated fatty acids in the cell membrane through its regulation of stearoyl-CoA desaturase to produce the mono-unsaturated fatty acid needed for membrane homeostasis and melanoma growth (Scott et al., 2022). Drug resistant cells can overcome this problem by increasing lipid uptake, beta-oxidation, phospholipid remodeling or through enhanced antioxidant defenses (Bacci et al., 2021). Thus lipid metabolism is not simply a consequence of phenotype switching; it determines whether melanoma cells grow, migrate or survive therapy or become susceptible to oxidative death.

Furthermore, within this metabolic network lipid droplets (LDs) are moving beyond being viewed as simple passive fat stores to being recognized as dynamic organelles that organize the intracellular fate of lipids (Renne and Hariri, 2021). They contain neutral lipid cores surrounded by a phospholipid monolayer containing proteins that regulate their biogenesis, growth, trafficking, lipolysis and organelle interactions. Lipid droplets receive fatty acids from the ER, exchange substrates with mitochondria and peroxisomes and undergo lysosomal degradation via lipophagy (Weiskirchen et al., 2026). These processes allow melanoma cells to store excess fatty acids away from toxic effects to the membranes due to lipid peroxidation, to generate energy through fatty acid oxidation, and to regulate lipid availability for membrane synthesis. However the functional impact of lipid droplets cannot be determined by their size alone. For example a droplet rich in monounsaturated lipids can protect membranes from oxidation while a droplet rich in polyunsaturated fatty acids can either store potentially damaging substrates away from reactive sites or release them once activated through lipolysis (Danielli et al., 2023). This context-dependent duality—protective storage versus pro-ferroptotic mobilization—is a recurring theme throughout this review.

These dual roles place lipid droplets at the interface between melanoma plasticity and ferroptosis. Ferroptosis is a non-apoptotic regulated form of cell death caused by uncontrolled iron dependent lipid peroxidation exceeding the cells’ capacity to repair oxidative damage (Liang et al., 2022). Ferroptosis requires oxidation prone membrane phospholipids, redox active iron and insufficient detoxification by glutathione peroxidase 4 or alternative antioxidant defenses such as ferroptosis suppressor protein 1 (Liu et al., 2022). Melanoma provides an ideal environment for studying ferroptosis given that many invasive and therapy-resistant states can increase oxidative stress while reducing lethal oxidative damage (Talty and Bosenberg, 2022). However this relationship is conditional: An MITF-low population may be ferroptosis prone if it contains large amounts of membrane polyunsaturated fatty acids but resistant if they are stored in neutral lipids, metabolized through beta oxidation, converted into less oxidizable phospholipids or protected by increased antioxidant defenses (Pellerin et al., 2020).

Current evidence indicates that ferroptosis sensitivities are not solely determined by individual molecular markers but are influenced by lipid organizational patterns (Naowarojna et al., 2023). Differences in ferroptosis onset among melanoma models were found to correlate with efficient sequestration of polyunsaturated fatty acids into lipid droplets and proximity of droplets to lipid modifying organelles (Kump et al., 2026). AMPK dependent modification of droplet mobility can thus modulate both the timing and amount of ferroptotic death. Inter-cellular lipid exchange adds another layer of complexity: ApoE secreted by MITF-high cells can decrease peroxidation-prone lipids in neighboring invasive cells while oleate rich lymphatic environments protect disseminated melanoma cells through ACSL3 mediated lipid remodeling creating conditions where FSP1 is clinically relevant (Motamedi et al., 2025).

Similarly total cellular iron is an imperfect indicator of ferroptosis potential since each pool has different effects on metabolism and radical generation. Changes in BDH2 dependent iron transport from lysosomes to mitochondria in transitioning from melanocytic to mesenchymal-like states can influence both cell-type plasticity and ferroptosis vulnerability (Key et al., 2020). Also cysteine availability can affect MITF expression and lysosomal function as well as the synthesis of glutathione thereby influencing the protection of lipid peroxidation. Collectively these findings demonstrate an interdependent relationship between lineage regulation, iron distribution and antioxidant defense within a single metabolic network (Huang et al., 2024).

Therefore therapeutic interventions require selective actions towards particular states. Systemic toxicity and compensation pathways make direct inhibition of GPX4 or cystine uptake ineffective against all ferroptosis-sensitized cells (Gagliardi et al., 2020). Greater selectivity can be achieved by disrupting the metabolic structure responsible for maintaining resistant melanoma below its ferroptotic threshold. Potential strategies include inhibiting DGAT-mediated lipid storage, suppressing SCD1-mediated desaturation, blocking ACSL3 or MBOAT1/2-mediated protective phospholipid remodeling, inhibiting fatty acid oxidation and manipulating organelle-specific iron trafficking. In BRAF inhibitor resistant melanoma fatty acid oxidation acts as a surveillance mechanism for ferroptosis while androgen-receptor controlled MBOAT1/2 activity prevents polyunsaturated fatty acid incorporation into membranes. Consequently resistant cells are concurrently primed for ferroptosis but protected from lethal peroxidation (Kump et al., 2026).

This article describes how lipid droplets serve as spatial determinants of metabolic vulnerabilities arising from melanoma phenotype switching. It argues that ferroptosis susceptibility cannot be predicted from MITF status, droplet number/size or antioxidant gene expression alone. Rather, ferroptosis susceptibility depends upon an integrated balance among seven cooperating determinants: fatty acid uptake, which controls the supply of oxidation-prone substrates; neutral lipid sequestration in lipid droplets, which buffers PUFAs away from membranes; lipolytic release, which re-exposes stored PUFAs; membrane phospholipid remodeling, which determines the abundance of oxidation-prone species; oxidative metabolism (β-oxidation), which consumes excess fatty acids; cysteine-dependent redox control via the glutathione–GPX4 axis; and organelle-specific iron availability, which determines where catalytic iron encounters PUFAs (Pellerin et al., 2020). Understanding this balance across various melanoma states can enable biomarkers for identifying treatment persistent populations with minimal toxicity. This connection between lineage plasticity and lipid droplet biology as well as ferroptotic control enables us to recognize organelle-based metabolic networks as a mechanistic explanation for therapeutic persistence as well as a source of new therapeutic vulnerabilities in advanced melanoma (Pisanu et al., 2026).

## 2 Melanoma phenotypic plasticity and the MITF low state

### 2.1 MITF as a regulator of melanocytic lineage identity

MITF controls all of the major aspects of melanoma development, from lineage specification and proliferation to pigment production and metabolic adaptation. As opposed to being simply a binary switch of cellular fate (i.e., either differentiated or undifferentiated), MITF coordinates a complex transcriptional program that varies by concentration and environment and determines a variety of biological endpoints (Gelmi et al., 2022). The traditional view has been that the primary roles of MITF are to control genes involved in melanin production, melanosomal maturation, lysosomal biogenesis, and cell cycle progression. Thus, changes in MITF activity can result in substantial differences in biological behavior without necessitating additional genetic mutations (Abrahamian and Grimm, 2021). MITF establishes a link between melanocytic lineage and mitochondrial metabolic processes. For example, in certain subsets of melanomas, MITF activates PPARGC1A/PGC-1α, resulting in enhanced mitochondrial biogenesis, increased rates of oxidative phosphorylation, and enhanced resistance to mitochondrial-derived oxidative stress (Huang C. et al., 2021). However, because these proliferative programs require high levels of mitochondrial respiration they likely will generate identifiable metabolic liabilities. Furthermore, MITF regulates redox homeostasis via a larger antioxidant transcriptional program including metabolic enzymes such as IDH1 and nicotinamide nucleotide transhydrogenase (NNT). These enzymes maintain NADPH stores and regenerate antioxidants necessary for reducing ROS. Therefore, MITF-high cells may be able to withstand elevated levels of oxidative stress due to their ability to coordinate mitochondrial and cytoplasmic antioxidant defenses (Roider et al., 2024). The developmental program established by MITF extends directly into the realm of fatty acid metabolism. MITF increases the expression of stearoyl-CoA desaturase (SCD) which is responsible for converting saturated fatty acids to monounsaturated fatty acids needed for membrane synthesis and lipid balance (Pellerin et al., 2020). The MITF-SCD regulatory axis impacts the level of unsaturation of fatty acids, which affects membrane fluidity, ER stress, inflammation-related signaling pathways, and overall melanoma phenotype. Disrupting this axis can decrease proliferation of melanomas that express high levels of MITF while simultaneously inducing ATF4-mediated stress responses and de-differentiation. Consequently, lipid composition is both an outcome of the action of MITF and a regulator of melanoma identity (Vivas-García et al., 2020).

### 2.2 Transition from proliferative to invasive melanoma phenotypes

The classical phenotype-switching model emerged from studies of melanoma cells which demonstrate that they are capable of alternating between a highly proliferative state which is characterized by less invasiveness and a more slowly proliferative state with increased motility and invasion (Hossain and Eccles, 2023). These findings challenged linear models of tumor progression whereby it was assumed that acquisition of increasingly aggressive mutations leads to irreversible increases in aggression. However, the findings demonstrated that melanoma cells can reversibly alter their transcription programs based on environmental conditions so as to permit different cellular states to perform complementary functions during growth and dissemination (Pagliuca et al., 2022).

MITF became the central element of the rheostat model. Low levels of MITF activity were initially associated with cell cycle arrest or senescence, intermediate levels of activity were associated with proliferation, while high levels of activity were associated with melanocytic differentiation (Lai et al., 2025). Subsequent research has demonstrated that the outcome of these processes are strongly modified by oncogenic signaling pathways, cellular stress responses and cooperating transcription factors. Therefore, proliferative populations are typically characterized by high levels of MITF and SOX10 activity, while invasive populations typically display reduced MITF activity accompanied by increased AXL activity, extracellular matrix remodeling, cytoskeletal reorganization and inflammatory signaling (Najem et al., 2022). The transition between these states is also reversible such that invasive cells may regain their ability to proliferate after entering a permissive metastatic environment.

Therefore, the distinction between proliferation and invasion is not absolute; MITF-low cells may retain proliferative competence while MITF-high populations may exhibit invasive behavior under certain microenvironmental conditions. Furthermore, drug-tolerant cells do not necessarily correspond to a single endpoint on the rheostat (Hossain and Eccles, 2023). Therapeutic stress can generate several slow-cycling or dedifferentiated populations each sustained through a different combination of lineage, metabolic, and stress response pathways. Although conceptually valuable, the MITF rheostat should be interpreted only as one dimension within a multidimensional landscape describing the cellular states that exist in melanomas (Fattore et al., 2020).

### 2.3 Contemporary melanoma cell state classification

Transcriptomic characterization combined with single-cell studies has created a continuum of semi-overlapping differentiation states from a previously described proliferative-invasive binary. One of the most commonly used models describes four states (melanocytic, transient, neural crest-like, and undifferentiated) (Belote et al., 2021). Cells that remain melanocytic retain high levels of MITF and SOX10 expression; expression of pigmentation programs; and lineage-specific metabolic characteristics. Transiently differentiated cells maintain many aspects of melanocytic cell identity while expressing initial stages of adaptive stress responses and de-differentiation pathways. Neural crest-like cells reactivate migratory, stem-like and environmentally responsive developmental pathways (Belote et al., 2021). Undifferentiated cells show diminished melanocyte marker expression and increased expression of inflammatory, mesenchyme-like, AP-1 associated and receptor tyrosine kinase gene sets. Additionally, these different states have varying sensitivities to iron dependent oxidative damage, thereby relating the differentiation state to ferroptosis related metabolism (Najem et al., 2022).

Therapeutic interventions generate additional state configurations. Analysis of minimal residual disease post-MAPK pathway inhibiting therapy found starved-like melanoma cells, neural crest stem like-cells, highly differentiated pigmented cells, and aggressive undifferentiated populations present in the same tumor (Liguoro et al., 2020). The starved-like program is thought to be an early adaptive response resulting from nutrient stress signaling and decreased proliferation potential. While cells are in the starved-like state, they may enter multiple drug tolerant paths instead of entering a predetermined sequential differentiation pathway. Neural crest stem-like cells are particularly relevant since they are resistant to MAPK inhibitors and will seed for subsequent resistance (Shah et al., 2026).

State designations should be viewed as continuous spectra and not as rigid taxonomic classification schemes. As such individual cells may reside in intermediate states; express markers designated for multiple states; and undergo transitions between the various programs over time (Wouters et al., 2020). State composition may vary significantly among the same lesion and between primary tumors, regional lymph node metastasis, and distant metastatic sites. Therefore, while MITF, AXL, and NGFR are useful markers they do not independently assess the entire spectrum of melanoma plasticity (Binder et al., 2021).

### 2.4 Molecular drivers of MITF suppression

MITF suppression may occur due to both microenvironmental and therapeutic convergence. The initial reduction in ERK-dependent proliferative signaling by the inhibition of the MAPK pathway in BRAF-mutated melanoma provides selective pressure to undergo transcriptional reprogramming (Proietti et al., 2020). Thus some cells may restore their ability to produce MAPK activity, whereas other cells lose their melanocytic character and express elevated levels of receptor tyrosine kinases, stress response regulators and developmental programs. Therefore, the lower MITF-to-AXL ratio has been correlated with decreased sensitivity to multiple targeted therapies; however, the MITF-to-AXL ratio is a state marker and not a causative process (Patel et al., 2021).

Signals generated through the TGF-β system are able to induce dedifferentiation through repression of melanocytic transcriptional programmes and induction of extracellular-matrix-, motility- and invasion-associated genes. Similarly inflammatory cytokines may cause a decrease in MITF expression and activation of signalling pathways including JAK–STAT, NF-kB or AP-1. Using chromatin analysis studies have shown that SOX10-MITF and AP-1-TEAD networks are two of the most important regulatory structures to influence proliferation/invasive states (Huang F. et al., 2021). TEADs have an effect on the invasive gene expression and also affect how tumors respond to inhibitors of the MAPK pathway. Additionally, the composition of AP-1 will determine the transcriptional environment which can either stabilize or assist in changing states (Mokhtari et al., 2023).

The adaptive programs identified by AXL and NGFR are overlapping but functional distinct. AXL is associated with receptor-tyrosine-kinase signaling, inflammatory activity, invasion and a wide range of drug resistance (Huang F. et al., 2021). NGFR defines neural crest like populations that are stress adapted and can survive during treatment, then repopulate the tumor. In addition to adaptive changes, hypoxia, nutrient deprivation, altered extracellular matrix stiffness, stroma-derived signals, mechanical stress can all contribute to programs that result in decreased MITF (Ortolano et al., 2026). Often these signals converge and act synergistically (for example matrix remodeling activates integrins and mechanotransduction pathways, while at the same time increases TGF-beta, AP-1 and TEAD activity).

### 2.5 Metabolic features of MITF low melanoma

Loss of lineage regulatory control due to loss of MITF results in heterogeneity in metabolic programming as opposed to uniform suppression. Loss of SCD (stearoyl Co-A Desaturase) could result in decreased endogenous synthesis of monounsaturated fatty acids, an increase in saturated fatty acid stress, and activation of ATF4 dependent adaptive pathways (Vivas-García et al., 2020). Cells lacking MITF could potentially have increased exogenous fatty acid uptake, altered elongation and desaturation pathways, or utilize available lipids for neutral storage, membrane remodeling, and energy production. Lipid profiles will vary based on what nutrients are present and what type of de-differentiated cell program that each individual cell adopts (Maresca et al., 2026).

The same thing is true about the mitochondria. Loss of MITF-PGC-1α can lead to reduced oxidative capacity in certain populations; however, cells with a neural crest-like program or those resistant to therapies may develop alternative fatty acid oxidation programs capable of sustaining both ATP levels and maintaining redox balance (Pellerin et al., 2020). It has been shown that models of melanomas in a melanocytic state can accumulate large amounts of lipid droplets, be heavily reliant on the oxidation of fatty acids for their energy needs, and demonstrate no apparent relationship between dedifferentiation and lipid droplet formation/fatty acid oxidation (Lumaquin-Yin et al., 2023).

Sulfur amino acid metabolism provides a connection between differentiation and the control of ferroptosis. Glutathione synthesis requires cysteine; however, it also maintains levels of MITF (melanocyte inducing transcription factor), acetyl CoA availability, chromatin acetylation, and lysosome function (Yu et al., 2024). Cysteine restriction leads to lower levels of MITF activity and promotes the switch from one cellular state to another via ATF4/NRF2 associated mechanisms. The direction and magnitude of the response to cysteine restriction will depend upon the resultant antioxidant capacity and lipid profile of the cells (Shah et al., 2026).

Finally, iron localization within a cell also changes during the process of dedifferentiation. State transitions from melanocytic to mesenchymal like states are accompanied by changes in lysosomal/mitochondrial iron pools versus increases in overall cellular iron (Rizzollo et al., 2025). Iron transfer from lysosomes to mitochondria mediated by BDH2 can provide a mechanism for state plasticity while increasing the potential for iron driven lipid peroxidation. Therefore, the potential for ferroptosis will depend on the location of the iron within the cell, the types of lipid substrate available, and whether or not sufficient antioxidants are available to neutralize the generated ROS (Key et al., 2020).

### 2.6 MITF low states in therapeutic persistence

Reversible dedifferentiation allows melanoma cells to survive targeted therapy without quickly acquiring new mutations that confer resistance. MAPK inhibition reduces proliferation that is lineage specific and enriches slow cycling cells that appear starved, neural-crest-like or undifferentiated (Hossain and Eccles, 2023). Cells survive through altered signaling of receptors, accessibility of chromatin, activation of stress responses and use of lipids. Once treatment is withdrawn or resistant signaling configuration emerges surviving cells can reenter the cell cycle and regenerate diverse tumors. Similar processes compromise blockade of immune checkpoints as well. Dedifferentiated melanoma can lose antigens that T cells recognize, reduce competence for antigen presentation and activate transcription programs that exclude T cells (Benboubker et al., 2022). Signals generated during an immune response paradoxically promote further dedifferentiation and create adaptive escape mechanisms where immune pressure changes target cell identity. Low MITF states also remodel cytokine production, extracellular matrix and metabolism of lipids and responses to oxidative stress weakening access or function of immune cells (Liang et al., 2026). Persistence of therapy then relies on both autonomous mechanisms within cells and ecological interactions. Low MITF cells can persist by adapting intrinsic metabolism and receiving lipids, cytokines, extracellular matrix signals or antioxidant support from neighboring melanoma, stromal cells and immune cells. Eliminating them will require interventions that take into account transitions that occur during treatment rather than relying solely on phenotyping beforehand (Sabău et al., 2025).

### 2.7 Heterogeneity within the MITF low phenotype

The relationship between dedifferentiation and increased sensitivity to ferroptosis has biological significance but remains incomplete. Loss of melanocyte-specificity as melanoma progresses will generally make melanocytes more susceptible to iron-dependent oxidative damage, especially those that have accumulated high levels of polyunsaturated fatty acid-containing membrane phospholipids and are deficient in GPX4-mediated protection (Ta et al., 2023). The state-of-differentiation analysis which identified transiently expressed, neural crest-like and undifferentiated melanomas demonstrated increased sensitivity of less differentiated states to ferroptotic conditions (Najem et al., 2022).

MITF-low cells can however develop multiple protective mechanisms against the initiation of ferroptosis. Increased fatty acid oxidation can utilize or redirect fatty acid substrates (for example, oleic acid) for energy or redox purposes (Maresca et al., 2026). DGAT-dependent formation of triglycerides from free fatty acids can encapsulate PUFA within lipid droplets thereby reducing the direct availability of these PUFAs to be incorporated into membrane phospholipids, where they would become oxidation prone (Motamedi et al., 2025). Use of MUFA by ACSL3 and remodeling of membrane phospholipids by MBOAT1/2 can reduce susceptibility of membranes to ferroptotic damage. Lastly, GPX4, FSP1, NRF2 and related systems provide layers of antioxidant surveillance to protect the cell (Yang et al., 2024). Of these, DGAT-mediated PUFA sequestration, ACSL3-dependent MUFA utilization and MBOAT1/2-mediated remodeling have been experimentally demonstrated in melanoma models, whereas their quantitative contribution to resistance in human tumors remains an emerging, hypothesis-driven question.

In BRAF inhibitor resistant melanoma, fatty acid oxidation and Androgen receptor mediated MBOAT1/2 activity can maintain cells that are otherwise poised metabolically for ferroptosis (Redondo-Muñoz et al., 2026).

Finally, in addition to the molecular heterogeneity described above there is a spatial component of heterogeneity due to differences in how PUFAs are organized into lipid droplets. The physical distance between lipid droplets and mitochondria and/or other lipid processing organelles influences the timing of ferroptosis (Motamedi et al., 2025). For instance, we hypothesize that two MITF-low populations with identical transcriptional profiles will behave differently depending on whether one stores its oxidizable lipids in relatively isolated neutral lipid pools or mobilizes them towards membranes or ROS-producing organelles; this hypothesis is consistent with AMPK-driven lipid-droplet redistribution toward mitochondria and its effect on ferroptosis timing (Motamedi et al., 2025).

Figure 1 integrates these observations into a dynamic model of melanoma-state progression (Hossain and Eccles, 2023).

**FIGURE 1:**
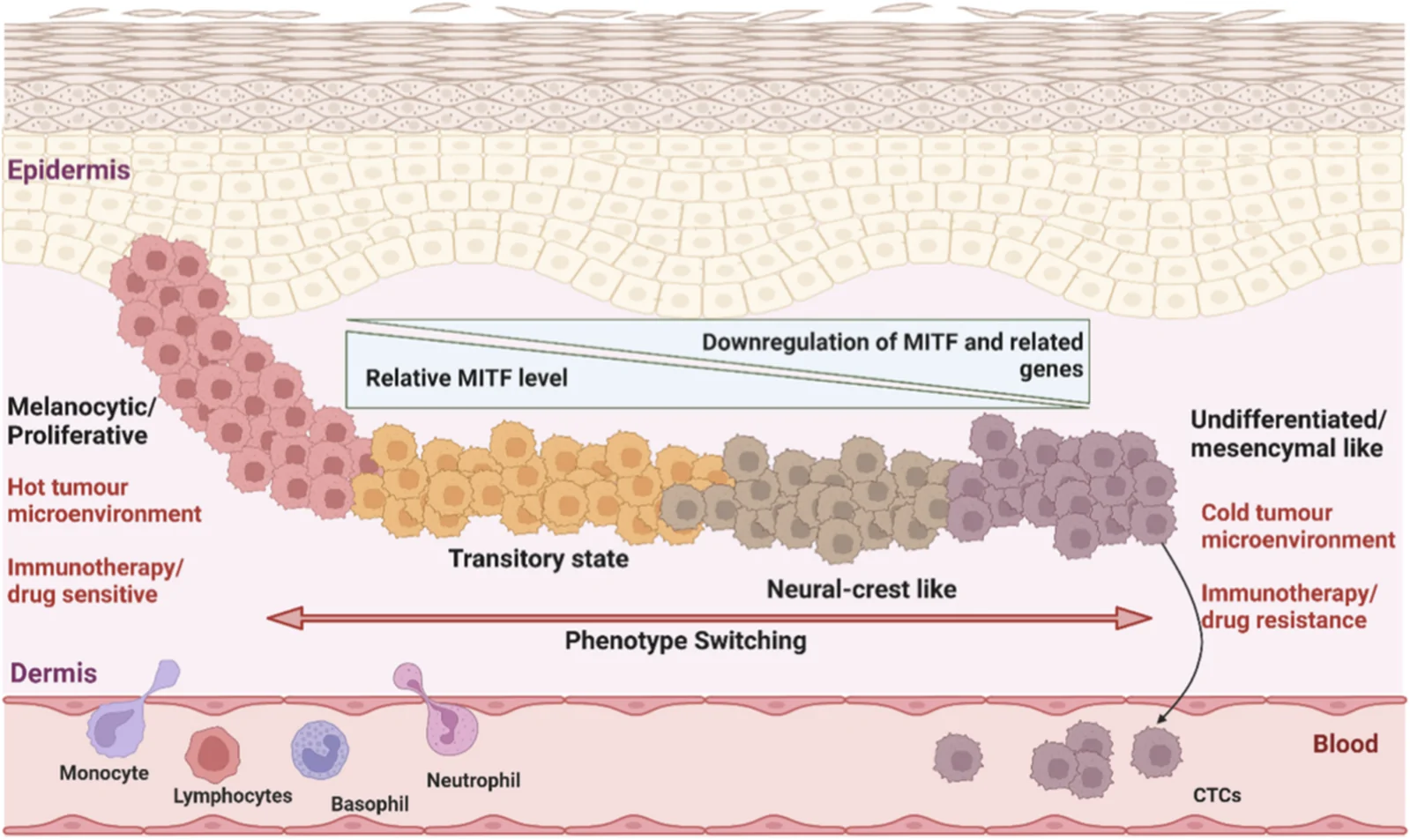
Depiction of phenotype switching during melanoma progression and the development of immunotherapy and drug resistance, based on the expression of MITFand MITF-related genes in the same tumor bed. Melanoma cells expressing low levels of MITF correspond to a slow-cycling and pro-invasive state (similar to “mesenchymal-like”), whereas higher levels of expression of MITF correlate with a proliferative and melanocytic state. Undifferentiated melanomas/neural-crest like (on the right side) melanomas lack activated immune cells, while melanocytic melanomas (left side) are composed of immunologically relatively more active immune cells/hot tumor microenvironment, which is also associated with melanocytic melanomas being relatively more responsive to immunotherapy or targeted drugs (Hossain and Eccles, 2023).

## 3 Lipid droplet biology in melanoma

### 3.1 Structural and molecular organization of lipid droplets

Lipid Droplets (LDs) are the site of accumulation of neutral lipids and have a unique structure. LDs contain a nonpolar interior (a “core”) primarily made up of triacylglycerols (TAG) and cholesteryl esters that are covered by a phospholipid monolayer decorated with perilipins and metabolic enzymes that regulate droplet stability, trafficking and lipolysis (Maresca et al., 2026). This surface proteome is a defining feature of LD structure and function (Olzmann and Carvalho, 2019). These properties distinguish LDs from bilayer-enclosed organelles such as mitochondria or peroxisomes. A single layer of phospholipids allows for the stable storage of TAG and other insoluble neutral lipids in the aqueous environment of the cell (Geltinger et al., 2020). Curvature, fusion, exposure to lipase activity and interactions with other subcellular compartments depend on the composition of this surface monolayer. Specifically, phosphatidylcholine (PC) appears to be critical for maintaining surface stability. An inadequate PC concentration can lead to aggregation and coalescence of LDs as well as altered availability of stored TAG (Weiskirchen et al., 2026).

Proteins that compose the proteome of LDs vary depending upon the stage of development, nutrient conditions and cellular requirements. In particular, members of the perilipin family are responsible for controlling access to the core of LDs where neutral lipids reside and serve as docking sites for enzymes involved in metabolism and for trafficking machinery (Boucher et al., 2021). For example, PLIN2 is typically found at mature cytoplasmic LDs and has been used to detect lipid-rich melanoma cells while PLIN3 is typically recruited to young developing LDs. Other proteins that may reside on the surface of LDs include acyltransferases, lipases, small GTPases, cytoskeletal adaptor proteins, ubiquitin-associated proteins, and proteins involved in tethering organelles together (Wei and Petritsch, 2026).

### 3.2 Lipid droplet biogenesis

Neutral lipid accumulation in the ER membrane forms the basis for the initial development of lipid droplets. Following their formation, as newly synthesized TAGs exceed their solubility on the ER membrane, neutral lipid molecules form an “oil-drop” like lens (Thiam and Ikonen, 2021). Continued lipid accretion onto this lens causes it to increase in size and create negative curvature (toward the cytosolic surface) of the endoplasmic reticulum. This process results in the emergence of a new lipid droplet from the ER membrane, yet maintains continuity through retention of a monolayer composed of phospholipid molecules from the cytosolic leaflet (Zadoorian et al., 2023).

The DGAT1 enzyme is primarily located within the lumen of the ER and is more effective than DGAT2 at converting diacylglycerols and fatty acids into TAGs. Although both enzymes can generate TAGs; however, they appear to be non-interchangeable (Zadoorian et al., 2023). While DGAT2 may also associate with ER-droplet interfaces and directly contribute to the enlargement of pre-existing droplets; DGAT1 appears to have a unique protective role against fatty acid induced-stress on the ER (Wei and Petritsch, 2026).

Following their formation, nascent lipid droplets continue to grow through local TAG synthesis and/or transfer of neutral lipids from the ER. Nascent lipid droplets may fuse/coalesce with neighboring droplets thereby increasing their overall size (Klemm and Carvalho, 2024). For this process to occur without excessive mechanical stress on the phospholipid layer that comprises the surface of each droplet, there must be coordinated expansion of the surface area of the droplet(s). Following droplet growth, recruitment of perilipin proteins and other lipid handling enzymes dictates whether the newly formed organelles will remain as large-scale TAG storage compartments, or serve as rapid sources of free fatty acids (Chandrasekaran et al., 2024).

### 3.3 Lipid droplet accumulation in melanoma progression

Lipid-droplet enrichment has been found in metabolically active melanoma populations, and there was an association with an increase in fatty-acid uptake and an increase in triacylglycerol production (Lumaquin-Yin et al., 2023). Experimental data were obtained for melanoma, where differentiated melanocytes had more lipid-droplets per cell than their less differentiated counterparts and required lipid storage and fatty acid oxidation (Maresca et al., 2026). Suppression of DGAT1 genetically or pharmacologically suppressed lipid-droplet formation, and suppressed cell cycle progression; however, DGAT1 suppression delayed tumor expansion *in vivo* when compared to tumors formed by non-suppressed melanomas. Thus, lipid-droplets may play a role in supporting melanoma growth post-tumor establishment (in addition to simply being indicative of lipid-exposure) (Lumaquin-Yin et al., 2023).

However, the relationship between droplets and aggressive behavior is likely to be non-linear. Droplets may allow for proliferation through providing membrane precursors and metabolic fuels, but they also provide mechanisms for survival during periods of nutrient depletion, oxidative stress, or pharmacologic insult (Xiong and Cheng, 2025). Melanoma cells are able to create a temporary delay between acquiring fatty acids and utilizing them through lipid-droplet formation. This delay allows cells to continue incorporating lipids into their metabolism without immediately having to commit those lipids towards membrane synthesis or oxidation. This delay provides adaptability to melanoma cells based upon changes in levels of oxygen, glucose, and/or extracellular lipid availability (Pellerin et al., 2020).

DGAT1 plays a significant role in facilitating stress-adaptation. The inhibition of DGAT1 results in increased oxidative stress within melanoma cells, resulting in enhanced reactive oxygen species-defense programs. There appears to be additive effects between inhibiting DGAT1 and antioxidants. The combination of both treatments resulted in greater tumor suppression than either treatment applied independently (Wilcock et al., 2022). The relevant content can be found in Table 1.

**TABLE 1:** Principal regulators of lipid-droplet metabolism and their relevance to melanoma.

Regulator	Core role in lipid-droplet metabolism	Relevance to melanoma	Predicted effect on ferroptosis	Translational relevance
DGAT1 (Motamedi et al., 2025)	Converts diacylglycerol into triacylglycerol and promotes lipid storage (Motamedet al., 2025)	Supports proliferation and oxidative-stress tolerance	Sequesters PUFAs away from membranes; stored lipids may later be released	Druggable target, particularly with GPX4 or antioxidant inhibition
DGAT2 (Wei and Petritsch, 2026)	Supports triacylglycerol synthesis and droplet expansion (Wei and Petritsch, 2026)	May compensate for DGAT1 loss	Delays PUFA membrane incorporation	Combined DGAT inhibition may increase efficacy but also toxicity
PLIN2 (Lumaquin-Yin et al., 2023)	Stabilizes mature lipid droplets and restricts lipase access (Lumaquin-Yin et al., 2023)	Marker of lipid-rich melanoma cells	Preserves fatty-acid sequestration	Potential biomarker of storage dependence
PLIN3 (Luo et al., 2022)	Stabilizes newly formed droplets and supports trafficking (Luo et al., 2022)	May facilitate adaptation to lipid influx	Promotes early sequestration of oxidizable lipids	Candidate marker requiring further validation
ATGL/PNPLA2 (Chen et al., 2021)	Initiates triacylglycerol hydrolysis (Chen et al., 2021)	Controls fatty-acid release and metabolic flexibility	May promote ferroptosis through PUFA release or support survival through oxidation	Strongly context dependent
ABHD5 (Cruz et al., 2020)	Activates ATGL-mediated lipolysis (Cruz et al., 2020)	Enhances lipid mobilization during stress	May increase oxidizable substrates while supplying metabolic fuel	Potential regulator of lipolytic dependence
HSL (Vasseur and Guillaumond, 2022)	Hydrolyses diacylglycerol during lipolysis (Vasseur and Guillaumond, 2022)	Supports sustained fatty-acid mobilization	Depends on whether released lipids enter membranes or oxidation	Limited melanoma-specific evidence
SCD1 (Maresca et al., 2026)	Generates monounsaturated fatty acids (Maresca et al., 2026)	Maintains membrane and lipid homeostasis	Reduces ferroptosis by increasing MUFA availability	Inhibition may sensitize SCD1-dependent tumors
ACSL3 (Kim et al., 2023)	Activates monounsaturated fatty acids (Kim et al., 2023)	Supports adaptation to lipid-rich environments	Promotes MUFA-mediated ferroptosis resistance	Candidate target in lymphatic or lipid-dependent melanoma
ACSL4 (Liao et al., 2022)	Activates long-chain PUFAs (Liao et al., 2022)	Promotes formation of oxidation-prone phospholipids	Increases ferroptosis competence	Useful biomarker of ferroptosis sensitivity
AMPK (Motamedi et al., 2025)	Coordinates energy stress, lipolysis, and droplet trafficking (Motamedi et al., 2025)	Regulates adaptation during nutrient deprivation	Can promote PUFA mobilization or support survival through oxidation	Effects depend on nutrient and organelle context
BECN1 (Lin et al., 2021)	Initiates autophagy and lipophagy (Lin et al., 2021)	Supports lipid recycling during stress	Can release stored PUFAs and influence ferroptosis	Limited by broad effects on autophagy
ATG5–ATG7 (Liu et al., 2025)	Enable autophagosome formation and droplet degradation (Liu et al., 2025)	Maintain nutrient recovery and organelle quality	May release oxidizable lipids or remove damaged material	Broad autophagy targeting may produce mixed effects
RAB7A (Liu et al., 2021)	Regulates lysosomal trafficking and lipophagic flux (Liu et al., 2021)	Supports lysosomal lipid recycling	Modifies lipid mobilization and iron availability	Promising but limited by selectivity concerns

### 3.4 Neutral lipid storage as a lipotoxicity control mechanism

Unesterified fatty acid molecules can break down lipid membranes, interfere with normal functioning of the endoplasmic reticulum, reduce mitochondria’s ability to produce ATP, and create toxic lipids as intermediate products (Smolková and Gotvaldová, 2025). The conversion of unesterified fatty acids to triglycerides provides a buffer against these potential biochemical problems by separating the acyl chain from water by trapping it in the center of the triglyceride droplet. All three types of fatty acids (saturated, monounsaturated and polyunsaturated) have this buffering capability. However, the biochemical results vary depending on the specific types of fatty acid being buffered (Smolková and Gotvaldová, 2025).

The storage of saturated fatty acids may limit the amount of endoplasmic-reticulum-stress promoting membrane composition available. Storage of monounsaturated fatty acids maintains a readily mobilizable pool of substrates for membrane synthesis and energy production (Maresca et al., 2026). Incorporation of polyunsaturated fatty acids into triglycerides will prevent them from directly entering phospholipids which can undergo oxidation. Therefore, triglyceride synthesis will determine both how many lipid molecules are present in cells at any one time and where the various molecular forms of each class of fatty acids go after they enter cells (Motamedi et al., 2025).

Although this protection provided by the presence of neutral lipid droplets has limitations. Droplets can never completely eliminate excess lipid molecules; additionally, all stored fatty acids are chemically reactive and thus metabolically active. Therefore, a lipid rich melanoma cell may survive an acute increase in fatty acid concentration and still retain a reserve pool that could be mobilized during periods of fasting or when subjected to some form of therapeutic stress (Bacci et al., 2021).

### 3.5 Lipid-droplet turnover and heterogeneity

Dynamic mobilization of lipid droplets occurs through cytosolic lipolysis and lysosome dependent lipophagy. ATGL (PNPLA2), catalyzes the initial hydrolysis of triacylglycerols and is activated by ABHD5 (Grabner et al., 2021). Subsequent breakdown of diacylglycerols is carried out by hormone sensitive lipase. Simultaneously, the autophagic machinery mediated by BECN1-, ATG5-and ATG7 delivers droplets or fragments of these droplets to lysosomes where lysosomal acid lipase is responsible for releasing the fatty acids stored (Yin et al., 2022). The two pathways may occur separately or concurrently depending on nutritional stress conditions. Once the lipid droplets are mobilized, they may be converted to energy through oxidation, reincorporated into phospholipids, secreted from cells or utilized for signaling purposes thereby influencing both metabolic adaptations and oxidative vulnerabilities (Pellerin et al., 2020).

Lipid droplets located within the same melanoma cell vary significantly in terms of their structural properties and functional capabilities. The droplets found in melanoma cells may differ based upon size, acyl chain composition, perilipin coating, mobility, subcellular location, and accessibility to lipolytic enzymes (Wei and Petritsch, 2026). For example, small lipid droplets may exhibit rapid transfer of lipids to the endoplasmic reticulum while larger droplets may offer greater storage potential but could potentially be less metabolically accessible. Droplets positioned at the periphery of the cell may intercept exogenous fatty acids while those localized near mitochondria, peroxisomes or lysosomes favor the synthesis of membranes, oxidation of the stored lipid or lipophagic degradation. These spatial relationships are influenced by changes in cellular nutrition, movement of components via the cytoskeleton and activation of AMPK. Therefore, total number of droplets or intensity of staining cannot be reliably used to accurately measure rates of lipid flow between organelles, rate of communication among organelles, and degree of ferroptosis susceptibility (Motamedi et al., 2025).

### 3.6 Lipid droplets as regulators of ferroptosis

The influence of lipid droplets on ferroptosis is dependent on the direction of lipid flow; i.e., how the lipid moves. When polyunsaturated fatty acids (PUFAs) are stored as triglycerides they cannot immediately be incorporated into PUFAs sensitive membrane phospholipids (Kim et al., 2023). Therefore, under normal conditions, lipid droplet formation will delay oxidative damage to cellular membranes. However, if there is interference with the formation of neutral lipids it may have an impact on this protective mechanism and whether or not it has an impact will depend upon where the displaced PUFAs go when they leave the droplet (Motamedi et al., 2025).

Alternatively, stored PUFAs can act as pro-ferroptotic substrates. For example, in melanoma cell lines, added PUFAs were primarily contained in triacylglycerols associated with droplets rather than being directly in membrane phospholipids. Cell line differences were noted in terms of time of death due to ferroptosis based on the location of the droplets and therefore the eventual release of PUFAs (Lee et al., 2024). Peripheral retention caused a delay in ferroptosis while nutrient depletion/AMPK activation promoted droplet movement towards mitochondria, hydrolysis of PUFA containing triglyceride, and transport of potentially oxidizable lipids to mitochondrial phospholipids. Reducing protein involvement in droplet movement or interaction between mitochondria and droplets both resulted in a reduction in lipid peroxidation and cell death (Motamedi et al., 2025).

Therefore, these studies demonstrate that lipid droplet formation confers no permanent resistance to ferroptosis. The same droplet can at first protect cells by reducing the number of PUFAs available for oxidation and subsequently contribute to oxidative damage by providing PUFAs to potential target organelle(s). Ultimately, the length of time that the PUFAs are stored, the energy status of the cell, how the PUFAs move around the cell, and whether or not redox active iron is present all determine what role will be most prominent (Kim et al., 2023).

### 3.7 Consequences of lipid droplet accumulation

Lipid droplet accumulation does not fully represent metabolic activity. High levels of lipid droplets might mean successful detoxification, active lipid incorporation, inhibition of lipolysis, poor autophagy degradation or readiness for oxidative metabolism (Pellerin et al., 2020). Low levels of lipid droplets could be due to insufficient fatty acid availability; rapid substrate utilization; inability to incorporate toxins into lipid droplets; etc. Each possibility carries with it significantly different implications for both tumor fitness and ferroptosis susceptibility (Lumaquin-Yin et al., 2023).

Thus, interpretation will require measurement of the compositional makeup (what type of fats), synthetic and degradative rates of lipid droplets as well as the spatial organization (where they reside) of lipid droplets. Using lipidomics, it can be established if the lipid droplets are composed primarily of saturated, monounsaturated or polyunsaturated triacylglycerols. Imaging live cells allows one to measure how rapidly lipid droplets turn over and interact with other cellular compartments. Assessing simultaneously membrane phospholipids and lipid peroxidation will allow one to determine if modifying the lipid content of lipid droplets protects against oxidative stress-induced death or increases the likelihood of such events.

## 4 Lipid droplet organized metabolic networks

We define a “lipid-droplet-organized metabolic network” as the integrated set of lipid uptake, storage, trafficking, remodeling, oxidation and antioxidant reactions whose spatial arrangement around lipid droplets determines whether a melanoma cell survives or undergoes ferroptosis. Melanoma cells use several ways to obtain fatty acids (the building blocks of fats) including receptor-assisted uptake, transporter mediated translocation across the cell membrane, lipoprotein processing, and passive diffusion. CD36 assists in transporting long chain fatty acids and oxidized lipids into the cells (Xu et al., 2021). Transport proteins facilitate both transport and intracellular acyl-CoA synthetase activities. After obtaining fatty acids, they associate with fatty acid binding proteins that prevent the fatty acids from diffusing out of the cell and help guide them to specific metabolic locations. Then the fatty acids are processed by acyl-CoA synthetases, acyltransferases, lipases and oxidative enzymes (Bacci et al., 2021). When converted to fatty acyl-CoA, it is now a “committed” intermediate that will have its own path to follow. Depending on what type of fatty acid it is, fatty acyl-CoA can be utilized for the formation of neutral lipids (triglycerides), phospholipid modification or β-oxidation. ACSL3 tends to utilize monounsaturated fatty acids in high fat environments while ACSL4 utilizes long chain polyunsaturated fatty acids that tend to be located in phospholipids prone to oxidation (Jiang et al., 2025). Oleic acid found in the lymph fluid can protect against oxidative death by directing ACSL3 dependent lipid trafficking in metastatic melanoma cells. Therefore, melanoma cells can take advantage of various types of lipid sources available in their environment to modify their lipid trafficking (Ubellacker et al., 2020).

The transport mechanisms described above also result in competitive relationships between various intracellular locations. For example, if a fatty acid is activated at the plasma membrane it is likely going to be transported into storage via storage pathways. Conversely, fatty acids activated near the ER or mitochondria are more likely to be involved in phospholipid synthesis or oxidation (Lumaquin-Yin et al., 2023). This allows melanoma cells to adapt quickly to changing environmental nutrients without having to change the amount of fatty acids available in their environment. Because these competing fates converge on or near the droplet surface, lipid droplets act as the organizing hub that routes each activated fatty acid toward storage, membrane remodeling or oxidation. Furthermore, the same concentration of lipids can have different effects depending on the combination of transport proteins and acyl-CoA synthetases present in the cells (Alkaraki et al., 2021).

When fatty acids enter the glycerolipid pathway, they are added sequentially to form lysophosphatidic acid, phosphatidic acid, diacylglycerol and eventually triacylglycerol. Both DGAT1 and DGAT2 perform the last esterification reaction creating a fork in the road that leads to either membrane synthesis or neutral lipid storage (Karagiota et al., 2022). What happens next is determined by the type of acyl chains that were sequestered. For example, the storage of saturated fatty acids can reduce endoplasmic reticulum stress; however, diverting polyunsaturated species from immediate incorporation into phosphatidylethanolamine and other oxidation sensitive membrane lipids can ultimately limit their ability to participate in lipid peroxidation (Maresca et al., 2026). The overall biological outcome is based on how much of the available fatty acyl-CoA is diverted to each location; the balance at this DGAT1/DGAT2 branch point determines how much fatty acid is retained within droplets versus diverted to membrane phospholipids. In melanoma cells, this provides a means of adapting to redox changes by physically segregating fatty acid acquisition from immediate membrane exposure (Peppicelli et al., 2024).

The ratio of monounsaturated to polyunsaturated fatty acids is one of the main factors determining the degree of stability exhibited by membranes. SCD1 converts saturated acyl-CoAs into monounsaturated products that can enter into triglycerides, phospholipids and cholesteryl esters (Pellerin et al., 2020). Monounsaturated chains contain fewer sites for free radical propagation compared to polyunsaturated chains; therefore, they can limit oxidative membrane injury. On the other hand, arachidonic, adrenic and similar polyunsaturated fatty acids constitute a substrate pool that could potentially be vulnerable to ferroptosis (Redondo-Muñoz et al., 2026). While they do not always lead to ferroptosis since they can remain in triglycerides or be metabolically oxidized for energy, susceptibility occurs when these fatty acids are activated and incorporated into membrane phospholipids at a rate greater than antioxidant mechanisms can repair the resulting peroxides. Oleate can mitigate the effects of decreased endogenous desaturation; therefore SCD1 expression alone is not sufficient to predict ferroptotic sensitivity without assessing lipid uptake and acyl chain distribution (Maresca et al., 2026).

In addition to selective phospholipid remodeling controlling which fatty acids occupy membrane positions, there exists a second level of regulation affecting which fatty acids are added to phospholipids. ACSL4 activates arachidonic and adrenic acids prior to incorporation into lysophospholipids by LPCAT3 thereby enriching membranes in species susceptible to peroxidation (Kim et al., 2023). Conversely, ACSL3 directs oleate usage and diminishes the contribution of highly oxidizable acyl chains when monounsaturated substrates are plentiful. MBOAT1 and MBOAT2 provide yet another layer of regulatory control over membrane composition through reacylation of lysophospholipids with selected fatty acids (Rodencal and Dixon, 2023). The presence of MBOAT1/2 in BRAF inhibitor resistant melanomas, along with the associated androgen receptor, results in membrane configurations that inhibit ferroptosis despite elevated levels of oxidative stress. Therefore, membrane composition is constantly regulated and remains responsive to treatments and nutrient status (Redondo-Muñoz et al., 2026). Because these remodeling enzymes draw on droplet-derived acyl pools, droplet composition directly constrains which fatty acids are available for membrane reacylation.

Physical and metabolic communication between lipid droplets and other organelles coordinates this process. Lipid droplets maintain continuous contact with the ER supporting neutral lipid synthesis, replenishment of droplet monolayers, and exchange of lipid-modifying enzymes (Wei and Petritsch, 2026). Triglycerides formed from stored fatty acids may return to the ER for additional phospholipid synthesis while newly synthesized triglycerides move away from the ER toward expanding droplet size. Acyl chains contained within triglycerides are generally shielded from oxidation occurring in membranes (Maresca et al., 2026). Alternatively, when re-introduced into endoplasmic-reticulum remodeling pathways, these acyl chains may serve as substrates for ferroptosis. Interfaces between endoplasmic reticulum and mitochondria may further localize phospholipid peroxidation by positioning lipid transfer and oxidative metabolism in close proximity (Zhuang et al., 2024).

Association with mitochondria permits controlled delivery of stored fatty acids for β-oxidation. Upon lipolytic release and activation, long chain acyl groups are transported into mitochondria through the carnitine shuttle generating acetyl-CoA, NADH and FADH_2_ (Vasseur and Guillaumond, 2022). Energy production can be sustained and excess fatty acids removed from potential vulnerabilities in membranes. However, increased respiratory flux can also increase generation of mitochondrial reactive oxygen species especially when substrate delivery exceeds oxidative capacity (Shen et al., 2020). Nutrient deprivation can activate AMPK leading to relocation of PUFA rich droplets toward mitochondria increasing transfer of oxidizable acyl chains into mitochondrial membranes. Therefore, proximity to mitochondria may enhance energy production in some cases but induce lipid peroxidation in others (Zhong et al., 2025).

Communication between lysosomes and lipid droplets integrate lipid recycling with iron homeostasis. During lipophagy autophagic structures deliver intact droplets or droplet pieces to lysosomes where lysosomal acid lipase breaks down neutral lipids releasing fatty acids. These fatty acids can be exported for oxidation, recycled for synthetic purposes or retained within the degradative compartment (Yang et al., 2023). Additionally lysosomes contain iron obtained from transferrin processing, ferritinophagy, damaged organelles and turnover of macromolecules. Co-localization of mobilizable lipids with redox active iron can enhance localized oxidation while retention of iron in lysosomes may prevent access to other compartments (Zhu et al., 2025). In melanoma cells, transfer of iron from lysosomes to mitochondria via BDH2 dependent mechanism alters ferroptotic sensitivity independent of total iron content.

Peroxisomes add an additional component to this network by processing very long chain and structurally complex fatty acids that cannot be easily imported into mitochondria. Shortening these molecules via peroxisomal beta oxidation can allow subsequent transfer back to mitochondria for complete oxidation or recycle back into synthetic pathways (Shen et al., 2020). Peroxisomes also play roles in PUFA metabolism and ether phospholipid synthesis. Ether lipids containing PUFAs can increase susceptibility to ferroptosis (Zou et al., 2020a) while peroxisomal oxidation itself produces hydrogen peroxide due to direct electron donation to O2. Catalase typically mitigates this oxidative burden; however imbalance between H2O2 production and detoxification can exacerbate cellular stress (Naowarojna et al., 2023). Peroxisomal ether-phospholipid synthesis also draws on fatty-acid pools that are supplied or buffered by lipid droplets, linking droplet composition to the abundance of oxidation-prone ether lipids.

Energy sensing via AMPK acts as a central regulator throughout this interconnected system. Low ATP combined with elevated AMP or ADP activates AMPK which suppresses energy intensive lipid synthesis and stimulates catabolic pathways. AMPK action at lipid droplets can affect droplet size, motility, access to lipases, as well as proximity to other organelles (Motamedi et al., 2025). During nutrient deprivation AMPK-mediated redistribution of PUFA-rich droplets toward mitochondria increases rates of fatty acid mobilization and ferroptic damage; however under other circumstances it may stimulate survival through stimulation of fatty acid oxidation and reduction of anabolic stress. As such its effect depends upon which type(s) of fatty acids are stored, which organelle receives them and the balance between substrate oxidation and peroxide detoxification (Smolková and Gotvaldová, 2025).

Adaptations made by melanoma cells during drug tolerant development exhibit shifts from dependency on glycolysis towards oxidative lipid utilization for energy production. Persister survival is supported through ACOX1 mediated peroxisomal oxidation while CPT1A regulates entry into mitochondria of long chain fatty acids sustaining ATP as well as maintaining redox homeostasis (Shen et al., 2020). Oxidation via mitochondria and peroxisome are complementary processes: peroxisomes shorten complex fatty acids while mitochondria extract remaining energy value. Disrupting only one of these compartments may redirect substrate flow towards the other compartment providing therapeutic benefits against targeted therapies and immunotherapies as well as removing surveillance mechanisms that limit ferroptosis in resistant melanoma (Liang et al., 2026).

Antioxidant mechanisms including cysteine/glutathione metabolism provide reductive counterbalance to lipid oxidation. System Xc(−) imports cystine into cells where it is reduced to cysteine used for glutathione synthesis. Glutathione then reacts with GPX4 converting phospholipid hydroperoxides into less reactive alcohols; continued protection requires NADPH-dependent glutathione regeneration provided by compartmentalized NADPH generating reactions including the pentose phosphate pathway and malic enzyme reactions (Kennedy et al., 2020). Depletion of cysteine can decrease repair capabilities for lipid peroxy radicals while eliciting adaptive programs that may produce either sensitization or resistance. Because droplets buffer PUFAs that would otherwise overwhelm these antioxidant systems, the size and composition of the droplet pool set the demand placed on glutathione/GPX4-dependent repair.

Iron is distributed among ferritin, heme-containing proteins, iron sulfur cluster-containing proteins, lysosomal stores, mitochondrial pools, and a small labile pool capable of participating in radical chemistry. Ferrous iron within the labile pool can initiate or propagate lipid oxidation; however total iron measurements cannot provide information regarding which membrane system contains redox active iron (Piccolo et al., 2024). Collectively, all components that regulate distribution include lysosomal release/uptake, mitochondrial import/export, transferrin uptake, ferritin stores/ferroportin-mediated export and iron responsive proteins. Ferroptotic injury becomes most probable when redox-active iron and PUFA-rich phospholipids colocalize within the same organelle/membrane contact site (Chen et al., 2021). These organelle-level relationships are summarized in Figure 2; the following section examines how the same network is differentially engaged across specific melanoma cell states.

**FIGURE 2:**
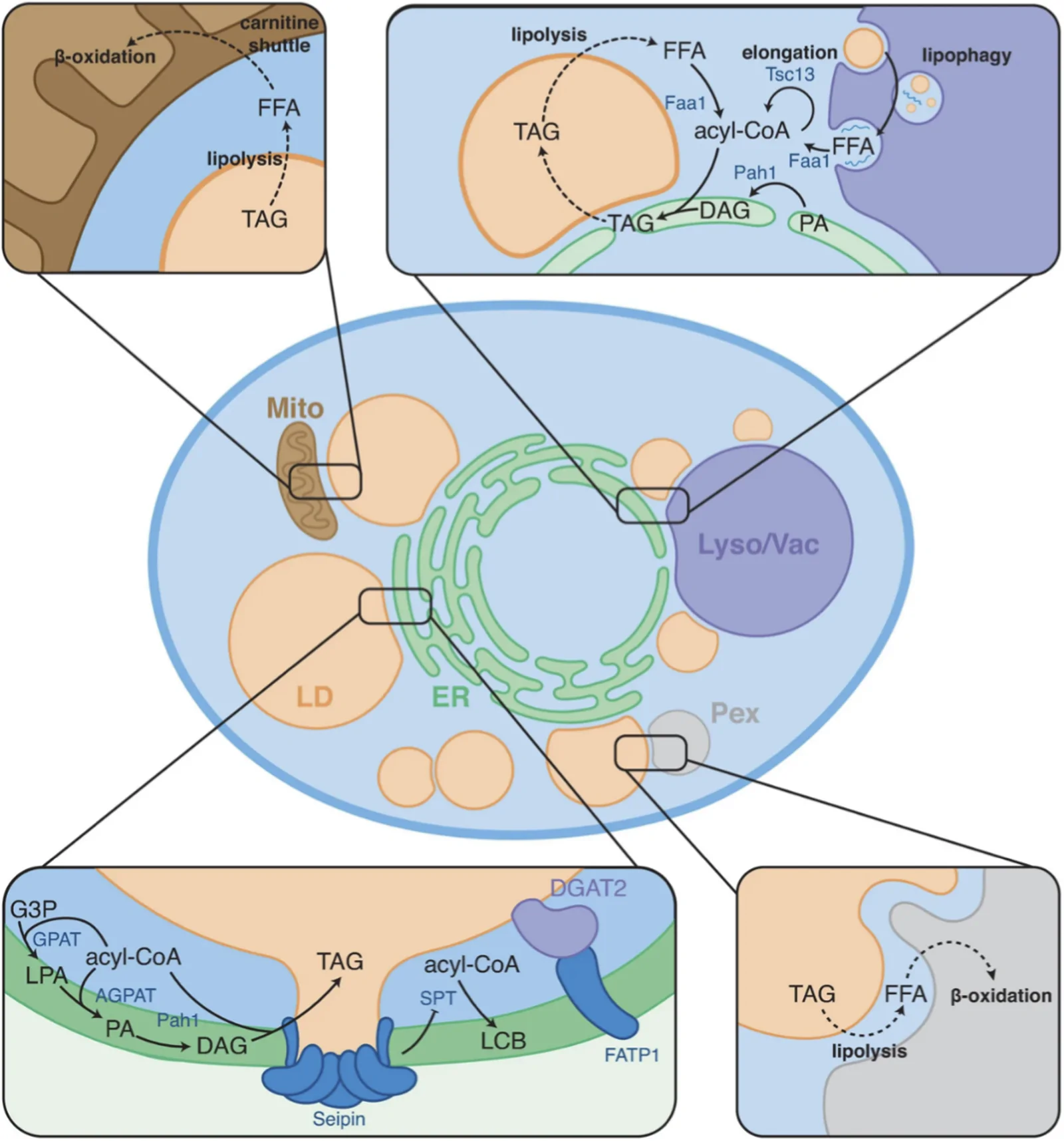
Overview of FA metabolic steps taking place at LD contact sites. LDs form contact sites with the endoplasmic reticulum (ER), mitochondria (Mito), peroxisomes (Pex), and lysosome/vacuole (Lyso/Vac). Boxes show examples of FA metabolic processes taking place at these contact sites. Arrows indicate enzymatic conversions; dashed arrows indicate trafficking/transport steps (Renne and Hariri, 2021).

Figure 2 illustrates integration of all processes illustrated previously showing that lipid droplets are positioned centrally within a directional network connecting extracellular uptake, triglyceride storage, phospholipid remodeling, organelle transfer, β-oxidation, iron traffic, and antioxidant repair (Renne and Hariri, 2021).

## 5 Ferroptosis regulation across melanoma cell states

Whereas the preceding section focused on the organelle-organized metabolic network, this section examines how ferroptosis is regulated across distinct melanoma cell states. In order to determine whether a given population of melanoma cells is likely to undergo ferroptosis it is essential to consider three key elements, the presence of lipid hydroperoxides; the requirement for redox-active iron to generate these hydroperoxides; the ability of radical-trapping antioxidants (such as ferrostatin-1) or iron chelators (such as deferoxamine) to prevent or reverse ferroptosis (Piccolo et al., 2024). The first element is demonstrated through biochemical assays such as those using ECL reagents, while the second is typically determined by demonstrating that inhibiting the activity of redox-active iron prevents ferroptosis. The third criterion is met if ferrostatin-1, deferoxamine, or another compound known to trap radicals or remove redox-active iron, respectively, reverses or prevents ferroptosis (Dixon et al., 2012).

ACSL4 selectively activates arachidonic and adrenic acids and other polyunsaturated fatty acids (PUFAs). These activated PUFAs are then incorporated into phosphatidylethanolamines and other membrane phospholipids via the action of LPCAT3. Activated PUFAs are prone to lipid peroxidation due to the presence of two bis-allylic hydrogen atoms. Redox-active iron catalyzes the formation of free radicals through nonenzymatic or enzymatic reactions (Kim et al., 2023). Enzymatically, arachidonate lipoxygenases (ALOXs)—in particular ALOX15 in complex with PEBP1—and cytochrome P450 oxidoreductase (POR) directly oxygenate PUFA-phospholipids to generate lipid hydroperoxides, thereby amplifying ferroptotic death signaling (Wenzel et al., 2017; Zou et al., 2020b).

Although high levels of ACSL4 and/or LPCAT3 mRNA indicate a potential to produce membranes that would be sensitive to lipid peroxidative damage, there are several factors that contribute to determining whether a particular population of cells will undergo ferroptosis. Antioxidant capacity, subcellular localization of iron and resultant phospholipid remodeling all play a role in determining whether a particular population of cells will undergo ferroptosis (Redondo-Muñoz et al., 2026).

Melanoma cells take up most of their iron from transferrin receptors located on their surface. While ferritin helps protect against reactive oxygen species by limiting radical chemistry through iron sequestration, once the available ferritin has been degraded, melanoma cells have limited options for preventing excessive production of free radicals (Plays et al., 2021). One option is ferritinophagy, where NCOA4 binds to ferritin and directs it towards lysosomes for degradation. When ferritinophagy occurs, the iron released from the ferritin can cause the formation of free radicals through Fenton type reactions. Alternatively, some of this released iron may be directed towards mitochondria, where it can contribute to oxidative damage to membrane lipids (Li et al., 2024).

Mitochondrial localized iron contributes to ferroptosis because it encounters phospholipids containing PUFAs. However, similar amounts of labile iron localized in lysosomes do not directly contribute to ferroptosis. Thus, redistribution of iron from lysosomes to mitochondria in melanoma cells contributes to alterations in both the plasticity of melanoma cells and their susceptibility to ferroptosis. Therefore, measuring compartmentalized iron content is more informative than total cellular iron content (Motamedi et al., 2025).

The major protective mechanism against lipid peroxidation involves a system consisting of cystine transporters SLC7A11 and SLC3A2, which facilitate cystine uptake into melanoma cells. Once inside, cystine is used for glutathione synthesis. Glutathione then acts as a reductant for GPX4, allowing it to convert phospholipid hydroperoxides into alcohol products that terminate oxidative chain reaction (Talty and Bosenberg, 2022). As a consequence, melanoma cells rely heavily on this pathway when they have high PUFA-phospholipid content, elevated labile iron or sustained oxidative activity. Disruption of this pathway through cystine deprivation, glutathione depletion, SLC7A11 inhibition or GPX4 blockade results in the induction of ferroptosis. It should be noted that compensatory mechanisms often make this disruption less effective (Piccolo et al., 2024).

In addition to supporting the GPX4 pathway, cysteine plays a role in maintaining melanocytic identity, regulating lysosome function and acetyl-CoA metabolism. Limitations in cysteine availability can thus lead to changes in both the ability to perform glutathione-based repairs and the likelihood of undergoing dedifferentiation under stress conditions. Ultimately, whether the transition to a MITF low-state leads to increased ferroptosis will depend on how rapidly and significantly the level of compensation mediated by NRF2 is achieved (Yu et al., 2024).

FSP1 exerts GPX4 independent protective effects by regenerating the radical-trapping antioxidant ubiquinol at cellular membranes. This could maintain viability in cells with partial GPX4 or glutathione deficiencies. This ubiquinol-regenerating activity may become especially important in lymphatic metastasis. DHODH maintains reduced coenzyme Q within mitochondrial membranes. Tetrahydrobiopterin formed by GCH1 activity, NRF2 regulated detoxification pathways and NADPH dependent enzymes represent additional forms of protection. These various mechanisms illustrate why increased lipid ROS does not always result in cell death (Li et al., 2025).

Cells differentiated in the MITF low-state exhibit characteristics that include decreased SCD1-dependent monounsaturated fatty acid synthesis, altered cysteine usage, inflammatory signaling and disordered iron distribution. All of these features potentially create higher basal oxidative pressures by increasing membrane polyunsaturations and bringing the cells closer to a state capable of producing sufficient lipid ROS to kill them. Therefore, a significant proportion of drug tolerant populations that survive apoptosis-inducing treatment with inhibitors targeting the MAPK pathway exist in a vulnerable state relative to ferroptosis due to their dependency on suppressive lipid hydroxide levels (Pellerin et al., 2020).

MITF high-states tend to be more oxidatively stable due to SCD1-generated monounsaturated fatty acids competing with polyunsaturated species for membrane incorporation thereby reducing the number of possible oxidation sites. Additionally, lineage associated glutathione and NADPH metabolism contribute to enhanced peroxide repair capabilities. These protections remain conditional since treatment with SCD1 inhibitors, cysteine availability modifiers, GPX4 inhibitors or FSP1 inhibitors can reveal previously latent vulnerabilities (Maresca et al., 2026).

Additionally, even within MITF-low-states there is heterogeneity regarding resistance to ferroptosis. Cells may employ mechanisms such as DGAT-mediated sequestration of PUFAs into triglycerides or fatty acid oxidation as means to evade ferroptosis. Other mechanisms include ACSL3-dependent utilization of extracellular monounsaturated lipids and MBOAT1/2-mediated phospholipid remodeling (Pellerin et al., 2020). For example, within BRAF inhibitor-resistant melanomas fatty acid oxidation and androgen-receptor-associated MBOAT1/2 activity help support cells that are otherwise poised to undergo ferroptotic priming. Survival in these circumstances is therefore based upon active metabolic protection rather than lack of susceptible substrate (Lee and Dixon, 2025).

Movement of lipid droplets also influences this response. Droplets located at peripheral areas of the cell can isolate PUFAs away from oxidative organelles such as peroxisomes and mitochondria. Conversely, droplet movement towards organelles such as mitochondria, peroxisomes, lysosomes or endoplasmic reticulum can enhance lipid peroxidation or lipid release into the cytoplasm. Subsequently, the droplet’s proximity to organelles containing redox-active iron can influence the rate at which PUFAs are converted into hydroperoxy radicals (Motamedi et al., 2025).

The tumor microenvironment also modulates many components involved in the regulation of lipid metabolism including redox status and mitochondrial biogenesis. Adipocytes provide fatty acids that modify membrane composition and support the activity of enzymes responsible for modifying fatty acids found within melanoma cells. For instance oleic acid stimulates the activity of ACSL3 leading to remodeling of mono unsaturated phospholipids in melanoma cells present within lymphatic tissue (Pellerin et al., 2020).

Hypoxic conditions combined with nutrient limitations in combination with cystine available in the external environment can also stimulate modifications in lipid metabolism and antioxidant capacity within melanoma cells. Moreover hypoxic conditions combined with stromal cytokines can also influence MITF expression and subsequently affect iron homeostasis within melanoma cells (Liang et al., 2026).

The process of metastasis selects for populations of melanoma cells that experience oxidative stresses sequentially throughout the process. For example, melanoma cells disseminated into circulation experience physical stresses as well as varying oxygen exposures and variable concentrations of soluble iron (Hong et al., 2021). Therefore populations with robust GPX4/FSP1/LPCAT3/ACSL3 defenses are selected for during early stages of dissemination. Conversely populations that colonize distantly located organs may lose their initial protective advantage once local lipid supply/oxygen concentration/iron homeostasis is disrupted (Kim et al., 2023).

## 6 Therapeutic exploitation of lipid organized vulnerabilities

Most lipid-directed ferroptosis strategies discussed below remain preclinical: DGAT, SCD1, ACSL3/4, MBOAT1/2 and fatty-acid-oxidation manipulations have been validated mainly in cell and animal models, and their clinical translation is limited by the availability of selective inhibitors and by systemic toxicity. Therapeutic use of lipid-based weaknesses in cancer will require coordination of access to the membrane substrate (lipid), antioxidants, and cell specific metabolic escape. Inhibiting System Xc–directly may help make melanoma cells rich in polyunsaturated fatty acid (PUFA) vulnerable to lethal phospholipid peroxidation; however, inhibiting either FSP1 or DHODH may be useful if there is coenzyme Q dependent protection at the plasma membrane or mitochondria as a result of GPX4 loss. The role of FSP1 is particularly evident in metastatic melanomas to regional lymph nodes as it presents a unique selective dependency on the local microenvironment different from the GPX4 dependency seen with hematogenous spread (Wang et al., 2026).

Metabolism of lipid droplets can provide an additional means to improve the specificity of therapy. Inhibiting DGAT1 or DGAT2 can inhibit the conversion of oxidizable fatty acids to triglyceride thereby increasing the amount available for incorporation into membranes. The greatest potential for inducing ferroptosis would exist under conditions in which PUFAs are unable to be removed via oxidation or redirected into other forms of storage (Motamedi et al., 2025). Both ATGL mediated lipolysis (the breakdown of triglycerides) and lipophagy (the degradation of lipid droplets) represent two approaches to regulate the release of fatty acids from pre-existing droplets. Increased turnover would potentially render PUFA loaded cells more sensitive to ferroptosis, whereas reduced mobilization may be beneficial if the released lipids are used to support oxidative metabolism and thus prolong treatment effectiveness (Alkaraki et al., 2021). DGAT1 inhibition has been shown to produce significant oxidative stress in melanoma suggesting its utility when combined with ferroptosis prevention strategies (Danielli et al., 2023).

The composition of membranes can also be modified by eliminating SCD1 generated monounsaturated fatty acids, by inhibiting ACSL3 dependent utilization of exogenously supplied oleate, or by preventing protective remodeling of phospholipids mediated by MBOAT1 and MBOAT2. Preserving both ACSL4 and LPCAT3 function should allow for maintenance of PUFA-phospholipid substrates necessary for ferroptotic execution (Kim et al., 2023). Evidence of alterations in lipid composition versus gene expression levels should guide selection of such interventions since the amounts of extracellular fatty acids and compensatory acyl transferase enzymes present can significantly affect final membrane composition (Pisanu et al., 2026).

Another possible targetable defensive mechanism against metabolically stable melanoma is fatty acid oxidation. Reducing mitochondrial and/or peroxisomal oxidation can limit ATP production, reduce the ability to remove mobilized fatty acids and enhance oxidative stress. Metabolic rewiring utilizing ranolazine was found to have improved efficacy of both targeted therapies and immunotherapies in experimental studies (Redondo-Muñoz et al., 2023). Recent studies have identified fatty acid oxidation and androgen receptor regulated MBOAT1/2 activities as cooperating mechanisms to evade ferroptosis in BRAF inhibitor resistant melanoma. There are several types of iron based strategies to promote lipid oxidation including ferritinophagy, lysosomal iron release, alteration of mitochondrial iron uptake/dumping and delivery of tumor specific iron. However, due to concerns regarding systemic iron toxicity, all of these strategies will need to be delivered in a space and time limited fashion (Kump et al., 2026).

Monotherapy with PUFA supplementation is unlikely to be successful as the administered lipids may be sequestered, oxidized, or otherwise detoxified prior to entry into susceptible membranes. Therefore, dietary, pharmacologic, or nanoparticle delivered PUFAs should be used in conjunction with DGAT, GPX4, FSP1, or inhibition of fatty acid oxidation. Additionally, the order of drug delivery is important and includes elimination of transitional or “starved” like cells as well as simultaneous disruption of oxidation, membrane remodeling, and peroxide repair in established MITF-low resistance states (Kump et al., 2026). Immune effector cells (such as CD8^+^ T cells) may further enhance this strategy by suppressing GPX4 function in tumor cells leading to ferroptosis; however, care will need to be taken to ensure redox homeostasis of immune effector cells is preserved. Several types of delivery systems (nanoparticles, antibody directed delivery vehicles, activatable prodrug delivery vehicles and localized delivery) can deliver high concentrations of these interventions to tumors while limiting damage to ferroptosis sensitive normal tissue (Tang et al., 2026). From a feasibility standpoint, the most clinically tractable near-term strategies are combinations of already-approved or repurposed agents with MAPK inhibitors or immunotherapy, whereas DGAT, MBOAT1/2 and iron-manipulation approaches will require biomarker-selected trials before clinical use.

The therapeutic relationships among melanoma state, metabolic dependency, and rational combination treatment are summarized in Table 2.

**TABLE 2:** Candidate therapeutic strategies for lipid-droplet-directed ferroptosis sensitization.

Therapeutic target	Principal mechanism	Predicted responsive melanoma state	Proposed biomarker	Rational combination	Major limitation
System Xc^−^ or GPX4 (Talty and Bosenberg, 2022)	Collapse phospholipid-peroxide repair (Talty and Bosenberg, 2022)	PUFA rich MITF low melanoma	SLC7A11, GPX4, lipid ROS	DGAT or FAO inhibition	Normal tissue toxicity
FSP1 or DHODH (Li et al., 2025)	Disable CoQ-dependent antioxidant defense (Li et al., 2025)	Lymph adapted or GPX4-independent melanoma	FSP1, CoQ redox status	GPX4 inhibition or immunotherapy	Context specific dependency
DGAT1 or DGAT2 (Motamedi et al., 2025)	Prevent protective PUFA sequestration (Motamedi et al., 2025)	Lipid droplet rich melanoma	DGAT activity, PUFA-triglycerides	GPX4 or FSP1 inhibition	Lipotoxicity and compensation
SCD1 or ACSL3 (Kim et al., 2023)	Reduce MUFA mediated protection (Kim et al., 2023)	MITF high or oleate dependent melanoma	SCD1, ACSL3, MUFA:PUFA ratio	PUFA delivery	Extracellular lipid rescue
MBOAT1/2 and FAO (Shen et al., 2020)	Block phospholipid remodeling and lipid disposal (Shen et al., 2020)	BRAF-inhibitor resistant melanoma	AR, MBOAT1/2, CPT1A, ACOX1	BRAF/MEK or GPX4 inhibition	Metabolic adaptation
Iron trafficking (Rizzollo et al., 2021)	Increase local redox-active iron (Rizzollo et al., 2021)	PUFA-rich, iron restricted melanoma	Ferritin and organelle iron pools	GPX4 inhibition	Systemic oxidative injury

Figure 3 integrates these treatment options into a biomarker-guided framework by separating MITF-high/SCD1-dependent, MITF-low/PUFA-rich, lipid droplet rich/DGAT-dependent, FAO-high drug-resistant, and ACSL3/FSP1-protected melanoma states (Ta et al., 2023).

**FIGURE 3:**
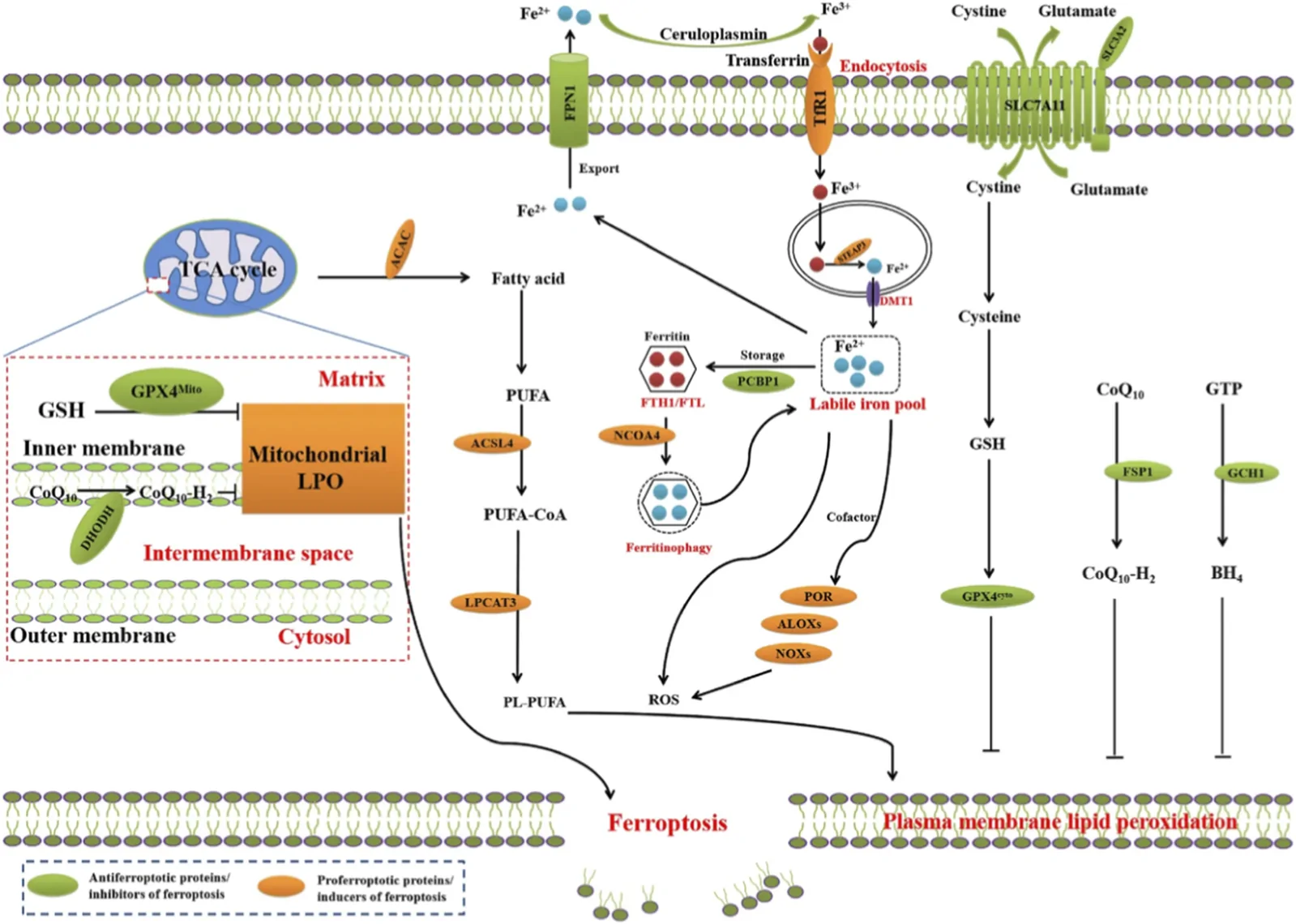
Core mechanisms of ferroptosis. The core of ferroptosis initiation is iron-dependent lipid peroxidation of polyunsaturated fatty acid (PUFA)-containing phospholipids (PUFA-PLs). When the ferroptosis-promoting factors (or Ferroptosis prerequisites) exceeding the buffering capability of cellular antioxidant systems (or ferroptosis defence systems), lethal accumulation of lipid peroxides on cellular membranes lead to membrane rupture, resulting in ferroptosis-related cell death. The ferroptosis-promoting factors consist of PUFA-PL synthesis and peroxidation, iron metabolism among others. Cells have evolved at least four ferroptosis defence systems, which includes GPX4/xCT system, the FSP1/CoQH_2_ system, the DHODH/CoQH_2_system, and the GCH1/BH_4_ system, with different subcellular localizations to detoxify lipid peroxides and thus protect cells against ferroptosis. The cytosolic GPX4 (GPX4^cyto^) cooperates with FSP1 on the plasma membrane (and other non-mitochondrial membranes) and mitochondrial GPX4 (GPX4^mito^) cooperates with DHODH in the mitochondria to neutralize lipid peroxides. ACSL4 and LPCAT3 mediate the synthesis of PUFA-PLs, which are susceptible to LPO through both non-enzymatic and enzymatic mechanisms. Iron initiates the non-enzymatic Fenton reaction and acts as an essential cofactor for ALOXs and POR, which promote LPO. When ferroptosis-promoting factors significantly exceed the detoxification capabilities of ferroptosis defence systems, an excessive and lethal accumulation of lipid peroxides on cellular membranes result in membrane rupture and trigger ferroptosis-mediated cell death (Ta et al., 2023).

## 7 Biomarkers and translational implementation

The need for composite clinical biomarkers (combining characteristics of melanoma cells; lipid composition and organelle structure; and sensitivity to programmed death) represents an important hurdle in translating lipid droplet directed ferroptosis strategies into a viable therapeutic option (Motamedi et al., 2025). Transcriptomic analysis will enable us to determine lineage and drug resistant states based on the levels of MITF, SOX10, AXL, and NGFR and also determine dependency on enzymes involved in ferroptosis including SLC7A11, GPX4, FSP1, ACSL3, ACSL4, DGAT1, SCD1, CPT1A, ACOX1, and MBOAT1/2. However, RNA levels are insufficient for accurately determining metabolite fluxes or membrane susceptibility especially when there are external influences on enzyme function via lipid availability in the blood and changes in enzyme activity due to post-translational modification (Deng et al., 2025).

Clinically relevant measurements will include the MUFA/PUFA ratio, relative amounts of oxidizable phospholipid species, triglyceride acyl chain composition, oxidized phosphatidylethanolamines, and secondary lipid peroxidation products. This information will allow to differentiate tumors which have inherently stable membranes against oxidation from tumors containing a large pool of ferroptosis susceptible lipids (Kim et al., 2023). This will help us understand if droplets are functioning as repositories of potentially toxic fatty acids or providing those fatty acids to oxidative compartments (Kump et al., 2026).

Finally, the determination of iron and redox status will add an additional level of classification. Thus, it will be necessary to measure cytoplasmic labile iron, ferritin abundance, transferrin receptor expression, localization of iron in lysosomes and mitochondria, glutathione levels, availability of NADPH, GPX4 activity, and redox state of CoQ. Since ferroptosis requires the spatial coordination of oxidation prone lipids, catalytically active iron and ineffective antioxidants, it is necessary to integrate all of these variables together (Deepak et al., 2026).

In addition to integrating these different types of data, we also believe single cell transcriptional studies and spatial transcriptional studies will aid in identifying small populations of drug tolerant cells that may be missed using traditional bulk analysis methods. Using mass spectrometry imaging and Raman microscopy will enable us to generate maps of lipid species across different areas of the tumor (Du et al., 2020). High resolution imaging studies of individual organelles will allow us to examine lipid droplet interactions with mitochondria, lysosomes, and the ER. Finally, we believe these technologies will also be useful in generating functional evidence demonstrating lipid ROS generation, requirement for iron, rescue by ferroptosis inhibitors, formation of oxidized phospholipids and lack of apoptosis or necroptosis (Motamedi et al., 2025).

As mentioned previously, validation studies will be conducted utilizing patient derived cultures, organoids, tumor slices, xenografted mice, syngeneic mouse models and longitudinally collected samples prior to treatment, during response and after relapse. We therefore propose a two-tier implementation: a minimal viable clinical panel (MITF/AXL/NGFR state markers; the MUFA/PUFA ratio and oxidized phosphatidylethanolamines; ferritin, transferrin receptor, GPX4 activity and the CoQ redox state) that can be deployed in routine diagnostic laboratories, and a research-grade comprehensive panel (single-cell and spatial transcriptomics, mass-spectrometry imaging, Raman microscopy, organelle iron mapping and dynamic fatty-acid tracing) for prospective validation cohorts. Once validated, pharmacodynamic assessments will be performed to evaluate how treatment affects membrane lipid composition, induces ferroptosis, alters melanoma cell states or selects alternative antioxidant and metabolic escape pathways (Lee and Dixon, 2025).

## 8 Limitations, challenges, and future directions

The application of translational research approaches to ferroptosis-related therapies in melanoma has been impeded by the unstable nature of tumor cell states. Metabolic programs of MITF, AXL, NGFR, and their related metabolic programs are subject to fluctuations depending on the location within the tumor, presence at metastatic locations, and stage of treatment. As such, a single biopsy does not represent sufficient information regarding the susceptibility of cancer cells to therapy. Similarly, the role of lipid droplets adds another layer of complexity due to the ability of these organelles to both protect against lipid peroxidation via storing polyunsaturated fatty acids (PUFA) and facilitate ferroptosis by releasing PUFA upon undergoing lipolysis, lipophagy, or being trafficked to other organelles. Therefore, the use of dynamic assessments are required to interpret the functionality of lipid droplets as opposed to static quantifications.

Experimental models commonly utilized in scientific study are unable to capture many aspects of human melanoma including cellular heterogeneity, interaction with immune cells, lymphatic conditioning, variability in lipid availability, and organ-specific metastatic microenvironments. Additionally, two-dimensional culture models have significant limitations when modeling lipid exchange and localized iron metabolism. Furthermore, validation of ferroptosis is often underutilized, with studies utilizing general reactive oxygen species (ROS), loss of viability without confirmation of phospholipid peroxidation, iron dependence, rescue by ferroptosis inhibitors, and exclusion of additional mechanisms of cell death. Additional limitation includes lack of standardization of criteria for defining melanoma state, lipid-droplet activity, levels of oxidized phospholipids, and dependency of ferroptosis. These limitations restrict the ability to compare results from different studies as well as accurately define subpopulations for future clinical trials.

A critical issue for developing safe therapeutics is the requirement of GPX4, SCD1, fatty acid oxidation and iron homeostasis in healthy tissues. Thus, broad inhibition may result in damage to neural tissue, liver, heart, kidney, or immune system. Treatment-resistant tumors may arise due to upregulation of lipid uptake, alternative desaturation pathways, switching energy sources or activation of antioxidant systems mediated by FSP1-, NRF2-, DHODH- and NADPH. Moreover, immunologic effects must also be considered since ferroptosis may increase presentation of tumor antigens whereas oxidative modification of lipids or non-specific redox injury may reduce lymphocyte functions.

Therefore, future studies should utilize multi-disciplinary approaches that include integration of single-cell and spatial RNA-seq data with lipidomics/metabolomics/proteomics and organelle imaging to differentiate ferroptosis-susceptible MITF-low populations from those that are metabolically protected. Use of stable-isotope tracing combined with live-cell microscopy will be crucial for tracking fatty acids from the point of external uptake through droplet storage/interorganellar transfer/membrane incorporation/lipid oxidation. In addition to the aforementioned methods, proximity labeling/contact site proteomics/super resolution microscopy and CRISPR screens will be useful for identifying regulators that control lipid exchange between lipid droplets/mitochondria/lysosomes/peroxisomes/endoplasmic reticulum.

Additionally, future clinical trials evaluating treatment timing should assess across three time points: early adaptation/minimal residual disease/resistance rather than assuming a consistent therapeutic window. Future precision clinical trials should select patients based on a combination of clinical state/lipidomic/iron/antioxidant biomarker status/paired biopsy specimens/pharmacodynamic evidence of lipid remodeling and ferroptosis engagement. Ultimately, this approach will provide the basis for converting metabolic vulnerabilities into a selective and clinically durable advantage.

## 9 Conclusion

Lipid droplets are central metabolic regulators in melanomas and connect cell identity, fatty acid partitioning, organelle communication and sensitivity to ferroptosis. Lipid droplets function beyond simply storing lipids; they regulate whether oxidative stress is buffered or enhanced through the direction of fatty acids toward triglyceride sequestration, membrane phospholipid synthesis, mitochondrial or peroxisomal oxidation and lysosomal recycling. The regulatory role of lipid droplets is particularly relevant in MITF-low melanoma where dedifferentiation results in altered lipid desaturation, redox control, iron trafficking and therapeutic persistence.

However, it should not be assumed that all MITF-low populations are equally sensitive to ferroptosis. Some populations may accumulate oxidation-prone phospholipids and become dependent on GPX4 (or related antioxidant systems) while others can maintain a degree of protection through dgat-mediated lipid storage, ACSL3-directed monounsaturated-fatty-acid utilization, MBOAT1/2 driven phospholipid remodeling, fatty acid oxidation or FSP1-supported radical suppression. Therefore, susceptibility to ferroptosis arises from the combined state of membrane composition, lipid droplet turnover, organelle proximity, antioxidant capacity and iron compartmentalization rather than from expression levels of MITF alone.

Therefore, strategies to exploit these vulnerabilities must include state resolved and biomarker-guided approaches. Direct induction of ferroptosis is likely to be most effective when combined with interventions designed to prevent lipid sequestration, restrict protective lipid remodeling, inhibit compensatory oxidation or increase tumor selective availability of iron. Combination with BRAF/MEK inhibition and immune checkpoint blockade will further eliminate drug-tolerant populations which persist following conventional treatment.

Ultimately, future progress will depend on combining spatial and single-cell profiling with functional lipidomics, dynamic fatty acid tracing, analysis of contact between organelles and rigorous validation of ferroptosis. Such approaches will permit precise identification of those cells within melanoma in which lipid droplets function as shields against metabolic damage and those in which they represent mobilizable sources of oxidative vulnerability. Targeting this distinction offers a rational path forward for overcoming resistance to therapy while improving the specificity of ferroptosis-based therapies.
